# Ruscogenin Attenuates Ulcerative Colitis in Mice by Inhibiting Caspase-1-Dependent Pyroptosis via the TLR4/NF-κB Signaling Pathway

**DOI:** 10.3390/biomedicines12050989

**Published:** 2024-04-30

**Authors:** Jingwei Li, Huihuan Wu, Jialiang Zhou, Rui Jiang, Zewei Zhuo, Qi Yang, Hao Chen, Weihong Sha

**Affiliations:** 1 Department of Gastroenterology, Guangdong Provincial People’s Hospital (Guangdong Academy of Medical Sciences), Southern Medical University, Guangzhou 510080, China; lijingwei@gdph.org.cn (J.L.); 202121050713@mail.scut.edu.cn (R.J.); zhuozewei@gdph.org.cn (Z.Z.); yangqi5056@163.com (Q.Y.); 2 Department of Gastroenterology, The Sixth Affiliated Hospital, School of Medicine, South China University of Technology, Foshan 528200, China; wuhuihuan@gdph.org.cn; 3 Department of Neonatal Surgery, Guangdong Women and Children Hospital, Guangzhou 511400, China

**Keywords:** ruscogenin, ulcerative colitis, pyroptosis, caspase-1, TLR4

## Abstract

Inflammatory bowel diseases (IBD) are chronic inflammatory disorders affecting the digestive tract, including ulcerative colitis and Crohn’s disease. Ruscogenin, a prominent steroidal sapogenin present in radix ophiopogon japonicus, has shown a protective effect on attenuating the inflammatory response associated with inflammatory diseases, but the efficacy of ruscogenin in IBD remains unclear. The aim of this study is to explore the effect of ruscogenin on intestinal barrier dysfunction and inflammatory responses as well as the underlying mechanism in ulcerative colitis. A dextran sulfate sodium salt (DSS)-induced C57BL/6 mouse colitis model was employed for the in vivo studies, while in vitro experiments were performed in THP-1 cells and human intestinal epithelial cells involved in inducing inflammatory responses and pyroptosis using LPS/nigericin. The results indicated that ruscogenin treatment attenuated the symptoms of ulcerative colitis, reduced the release of inflammatory cytokines and the expression of pyroptosis-associated proteins, and restored the integrity of the intestinal epithelial barrier in colon tissue in mice. Moreover, ruscogenin inhibited LPS/nigericin-induced pyroptosis in THP-1 cells. Mechanically, ruscogenin inhibited NLRP3 inflammasome activation and canonical pyroptosis, at least in part, through the suppression of the TLR4/NF-κB signaling pathway. These findings might provide new insights and a solid foundation for further exploration into the therapeutic potential of ruscogenin in the treatment of IBD.

## 1. Introduction

Inflammatory bowel diseases (IBD) are chronic inflammatory disorders affecting the gastrointestinal tract, with ulcerative colitis (UC) and Crohn’s disease being the two primary subtypes [1,2]. These conditions are characterized by recurring episodes of inflammation in the intestines, leading to various debilitating symptoms and impaired quality of life for affected individuals. The incidence and prevalence of IBD in the newly industrialized countries in Asia, the Middle East, and South America have steadily increased over the past decade [3]. It is estimated that an ageing IBD population will bring new challenges and complexities to gastroenterology clinics in the world in 2025 [4]. The etiology of IBD remains complex and multifactorial, but researchers have recently confirmed that dysregulated immune responses, defects in the intestinal epithelial barrier, and altered cell death processes play vital roles in the pathogenesis of these diseases [5]. To date, the main drug for the treatment of IBD include 5-aminosalicylates, corticosteroids, immunosuppressive drugs, and biological drug targeting at cytokines. Nevertheless, the prohibitive cost of monoclonal antibodies and the potential side effects associated with corticosteroids and immunosuppressive drugs impose limitations on their long-term utilization [6]. The development of novel drugs and improvement of the treatment strategy by implementing personalized medicine are warranted to achieve optimal disease control. Therefore, the development of potential innovative strategies for IBD treatment is highly urgent.

Pyroptosis is a highly regulated form of programmed cell death, which is a physiological response to host defense against microbial infections and tissue injury [7]. Pyroptosis plays an vital role in the occurrence and progression of various inflammatory diseases such as IBD [8,9]. The activation of pyroptosis involves different caspases, the most significant of which is the canonical pyroptosis pathway dependent on caspase-1 [10]. In the canonical pyroptosis pathway, the activation of the NLRP3 inflammasome recruits ASC and caspase-1 precursors and leads to subsequent activation of caspase-1. On the one hand, activated caspase-1 cleaves GSDMD to produce the N terminus, which binds to the cell membrane to form a membrane pore, leading to cell rupture. At the same time, activated caspase-1 cleaves and activates the inflammatory cytokines IL-1β and IL-18, which are then released to the outside of the cell through the membrane pores to cause inflammation. Moderate pyroptosis eradicates pathogenic microorganisms and induces tissue repair and slowly subsides after the removal of foreign bodies or repair of tissue damage. The vicious circle of dysregulated cell death, intestinal barrier destruction, and consequent inflammation lies at the core of chronic inflammatory diseases [11]. Thus, the accurate regulation of pyroptosis is a potential therapeutic strategy for IBD.

The Toll-like receptors 4 (TLR4) pathway is a key player in pyroptosis [12]. When cells are exposed to exogenous or endogenous stimuli, the pattern recognition receptors (PRRs) on the cell membrane activates TLR4 by recognizing pathogen-associated molecular patterns (PAMPs) or damage-associated molecular patterns (DAMPs) released by body cells. TLR4 promotes the transfer of nuclear transcription factor NF-κB into the nucleus through the MyD88 pathway. Then, NF-κB can not only promote the expression of NLRP3 to participate in the regulation of inflammasomes but also promote the expression of various pro-inflammatory genes, including inflammatory cytokines and chemokines [13]. Therefore, TLR4/NF-κB signaling pathways play an important role in regulating inflammatory responses and pyroptosis [14].

Given the complexity and limited treatment options for IBD, researchers have turned their attention to herbal medicine as potential interventions [15]. Ruscogenin, a prominent steroidal sapogenin present in radix ophiopogon japonicus, has emerged as a promising candidate for attenuating the inflammatory response associated with inflammatory diseases and cardiovascular diseases [16]. Notably, a previous study uncovered that ruscogenin has a protective effect on lung epithelial injury induced by LPS, and the possible mechanism is related to the inhibition of the NF-κB signaling pathway [17]. Moreover, recent studies have shown that ruscogenin alleviates particulate acute lung injury in mice by protecting the pulmonary endothelial barrier and inhibiting the TLR4 signaling pathway [18,19]. Another study found that ruscogenin attenuates cerebral ischemia-induced blood–brain barrier dysfunction by inhibiting NLRP3 inflammasome activation [20]. However, to date, there is a dearth of literature documenting the utilization of ruscogenin as a therapeutic intervention for IBD. In addition, the efficacy and mechanism of ruscogenin in ameliorating dextran sulfate sodium salt (DSS)-induced colitis by mitigating the extent of pyroptosis through regulating TLR4/NF-κB signaling pathway remains unclear and deserves further investigation.

In summary, we hypothesize that the administration of ruscogenin can attenuate DSS-induced colitis by improving intestinal barrier function, reducing the inflammation response and regulating pyroptosis via TLR4/NF-κB signaling pathway. The main purpose of this study is to elucidate the specific mechanism of ruscogenin in treating DSS-induced colitis model. The results of this study will provide promising treatment options and theoretical basis for the treatment of IBD patients.

## 2. Materials and Methods

### 2.1. Animal Experiments

SPF male C57BL/6 mice aged 6–8 weeks (weight 18–25 g) were purchased from Guangdong Province Animal Experiment Center. The mice were acclimated in their cages for a week before the experiments were conducted. The animals were maintained in standard condition at 23 ± 2 °C and (50 ± 10) % humidity, with a 12/12 h light and dark cycle and free access to water and food. All animal care and experiments were carried out in accordance with institutional animal care and use committee guidelines, and the procedures were approved by the ethics committee of Guangdong Provincial People’s Hospital, Guangzhou, China (approval number: KY-N-2022-084-02; approval date: 30 August 2022).

### 2.2. Chemicals and Reagents

Ruscogenin (Rus, purity 99%; chemical formula: C27H42O4; molecular weight: 430.62) was purchased from J&K Scientific Ltd. (San Jose, CA, USA). Dextran sulfate sodium salt (DSS, molecular weight: 36,000–50,000) was purchased from MP Biomedicals (Irvine, CA, USA). Dulbecco’s modified eagle medium (DMEM), RPMI 1640 medium, and fetal bovine serum (FBS) were purchased from Gibco (Carlsbad, CA, USA). LPS was purchased from Sigma-Aldrich (St. Louis, MO, USA). Nigericin was purchased from Invitrogen (Carlsbad, CA, USA). Overexpression plasmids for Flag-tagged TLR4 (pLV3-CMV-TLR4(human)-3×Flag-EF1a-CopGFP-Puro) and control plasmid were purchased from Miaoling Biology (Wuhan, China). The primary antibodies using in Western blotting were purchased from Abcam (Cambridge, UK) and Cell Signaling Technology (Boston, MA, USA), and the HRP-conjugated anti-rabbit and anti-mouse IgG antibodies were purchased from Cell Signaling Technology (Boston, MA, USA). The primary antibodies and the appropriate fluorescence conjugated secondary antibodies used in immunofluorescence staining were purchased from Abcam (Cambridge, UK).

### 2.3. DSS-Induced Colitis Model and Animal Experiment Design

The SPF male C57BL/6 mice were randomly divided into five groups. The mice in the control group (Ctrl) were treated with drinking water. The mice in the DSS model group (DSS) were administered drinking water that contains 3.5% DSS for 7 days. The mice in the Rus 0.5 mg/kg group (Rus 0.5) were administered drinking water containing 3.5% DSS for 7 days, followed by 0.5 mg/kg ruscogenin treatment by gavage daily. The mice in the Rus 5 mg/kg group (Rus 1) were administered drinking water containing 3.5% DSS for 7 days, followed by 1.0 mg/kg ruscogenin treatment by gavage daily. The mice in the Rus 2 mg/kg group (Rus 2) were administered drinking water containing 3.5% DSS for 7 days, followed by 2.0 mg/kg Ruscogenin treatment by gavage daily. Animals were monitored daily by weight and disease activity index score. After 7 days, the drinking water was switched to normal water for 1 day, and all the mice were euthanized on day 8. The serum and colon tissues samples were collected for subsequent analysis. The colon was excised from the cecum to 2 cm above the anus. The length of the colon was measured, which indirectly specified the inflammatory index of the colon.

### 2.4. Assessment of Disease Activity Index

To evaluate the disease activity index (DAI) of the mice in each group, the body weight, feces shape, and gross rectal bleeding of the mice were recorded and scored, according to the previous study [21]. In short, (1) loss of weight: less than 1%, score 0; less than 5%, greater than 1%, score 1; less than 10%, greater than 5%, score 2; less than 20% but greater than 10%, score 3; more than 20%, score 4; (2) feces shape: normal shape, score 0; loose stools score 1; liquid stools, score 3; and diarrhea score 4; (3) gross rectal bleeding: normal, score 0; mild bleeding, score 2; obvious bleeding, score 4.

### 2.5. Histological Evaluation

After 8 days, the mice were euthanized, and the colon tissues of each group were collected. The colon tissues were fixed with 10% formalin and embedded in paraffin. Then, colon tissues were cut into slices and stained with hematoxylin and eosin (H&E). The histology score was determined by multiplying the percent involvement of each of the five histological subscores [22]. For each parameter (0, absent; 1, mild; 2, moderate; 3, severe): mononuclear cell infiltrate (0–3), epithelial injury/erosion (0–3), crypt hyperplasia (0–3), polymorphonuclear cell infiltrates (0–3), and transmural inflammation (0–4). Extent factor was derived according to percent area involvement: 0, 0%; 1, <10%; 2, 10–25%; 3, 25–50%; and 4, >50%.

### 2.6. Cell Culture

Human acute monocytic leukemia THP-1 was purchased from Cell Bank of the Chinese Academy of Sciences (Shanghai, China). THP-1 cells were cultured in RPMI 1640 medium, supplemented with 10% FBS and 0.05 mM β-mercaptoethanol. Human intestinal epithelial cells (HIEC) and HEK-293T cells were purchased from Cell Bank of the Chinese Academy of Sciences (Shanghai, China) and were maintained in DMEM with 10% FBS. Cells were cultured in a humidified environment with 5% CO_2_ at 37 °C. Differentiation of THP-1 cells was induced by 0.5 mM phorbol 12-myristate 13-acetate (PMA, Sigma Aldrich, St. Louis, MO, USA) for 3 h. The differentiated cells were washed three times with PBS. In this study, cells were seeded in 6-well plates at a density of 5 × 10^6^ cells per well overnight. THP-1 cells were stimulated with LPS and nigericin to activate NLRP3 inflammasomes to mimic inflammatory environment in vitro. First, the cells were pre-treated with DMSO or different concentrations of ruscogenin for 24 h (2.5 μmol/L, 5 μmol/L, and 10 μmol/L), then treated with 100 ng/mL LPS, or left untreated for 4 h in serum-free medium, followed by treatment with 1 μM nigericin (Invitrogen, Carlsbad, CA, USA) for 4 h, as previously described [23]. The experimental groups for this study were defined as follows: Ctrl: normal control group; LPS: 100 ng/mL LPS exposure for 4 h and followed by treatment with 1 μM nigericin for 4 h; Rus 2.5 μM: 2.5 μM ruscogenin pretreatment for 24 h prior to LPS exposure for 4 h, followed by treatment with 1 μM nigericin for 4 h; Rus 5 μM: 5 μM ruscogenin pretreatment for 24 h prior to LPS exposure for 4 h, followed by treatment with 1 μM nigericin for 4 h; Rus 10 μM: 10 μM ruscogenin pretreatment for 24 h prior to 100 ng/mL LPS exposure for 4 h, followed by treatment with 1 μM nigericin for 4 h. For TLR4 overexpression experiment, normal THP-1 cells were treated with LPS/nigericin or ruscogenin (10 μM). THP-1 cells transfected with TLR4-plasmid were treated with LPS/nigericin and ruscogenin (10 μM). In brief, DMSO or 10 μM ruscogenin pretreatment for 24 h prior to 100 ng/mL LPS exposure was followed by treatment with 1 μM nigericin for 4 h.

### 2.7. Cell Viability Assay

The effects of ruscogenin on THP-1 cells was measured by using the cell counting kit-8 (CCK-8) assay (Guangzhou Bin Peng Biotechnology, Guangzhou, China). In brief, THP-1 cells were seeded in 96-well plates at 5 × 10^4^ per well overnight. Cells were treated with complete medium containing ruscogenin (0–320 μM) for 24 h, and then, 10 μL of CCK-8 reagent was added to each well and incubated for 2–3 h at 37 °C. The optical density (OD) value at 450 nm was detected using a microplate reader.

### 2.8. Cell Transfection

To further clarify the potential molecular mechanism of ruscogenin in inhibiting LPS/nigericin-primed pyroptosis, HEK-293T cells were transfected with Flag-TLR4 plasmid and control plasmid using Lipofectamine 2000 (Invitrogen, Carlsbad, CA, USA), and then, the culture medium was changed to normal complete medium after 4–6 h. THP-1 cells were cultured in 6-well plates. After 48 h and 72 h, viral fluids were obtained and used to infect THP-1 cells. After transfection, puromycin was used to select successfully transfected THP-1 cells.

### 2.9. ELISA

The levels of IL-6, TNF-α, MCP-1, and IL-18 in the colon tissue of the mice and the collected cell culture media were detected by ELISA kits (Proteintech, Wuhan, China; Cusabio, Beijing, China; Beyotime Biotechnology, Shanghai, China). The assay followed the instruction manual.

### 2.10. Quantitative Real-Time Polymerase Chain Reaction (qPCR)

Total RNA were extracted from colon tissues and cells using the Total RNA Isolation Kit (Vazyme, Nanjing, China). Reverse transcription was carried out using the Prime Script TM RT-PCR Kit (Takara, Otsu, Japan). Then, qPCR was performed with the Biorad CFX Connect (Biorad, Hercules, CA, USA) using the ChamQ Universal SYBR qPCR Master Mix (Vazyme, Nanjing, China), according to the manufacturer’s instructions. The 2^−ΔΔCt^ method was used to calculate the relative expressions of mRNAs, and the levels of the target genes were normalized to that of the *GAPDH* gene. Primer sequences used in this study are listed in Table S1.

### 2.11. Western Blotting

The Western blotting was carried out exactly as previously described [24]. The total proteins were extracted from mice colon tissues and cells as well as supernatant with RIPA lysis buffer containing protease and phosphatase inhibitor (Beyotime Biotechnology, Shanghai, China). The protein concentrations were determined with an BCA protein assay kit (Leagene Biotechnology, Beijing, China). The samples were prepared in 4× sodium dodecyl sulfate–polyacrylamide gel electrophoresis (SDS-PAGE) sample loading buffer (Invitrogen, Carlsbad, CA, USA). Protein samples were subjected to SDS-PAGE and transferred to PVDF membranes. After incubation with 5% non-fat milk, the membranes were incubated with indicated primary antibodies at 4 °C overnight. Then, the membranes were washed with TBS containing 0.1% Tween-20 (TBST) three times and then incubated with the HRP-conjugated anti-rabbit or anti-mouse IgG antibodies for 2 h at room temperature. Finally, the membranes were washed three times with TBST, the protein bands were visualized using an ECL Western Blotting Substrate (Biosharp, Anhui, China), and the signals were recorded with the Image Quant LAS 500 System (Cytiva, Marlborough, MA, USA). The results were quantified using Image J 1.8.0 software (http://rsb.info.nih.gov/ij/, accessed on 1 February 2024) and normalized to GAPDH or Tubulin. The antibodies used in this study are listed in Table S2.

### 2.12. Immunofluorescence Staining

Cells were seeded in confocal dish at a density of 5 × 10^3^ cells overnight. The cells were pre-treated with DMSO or ruscogenin (2.5 μM, 5 μM, and 10 μM) for 24 h, then treated with 100 ng/mL LPS, or left untreated for 4 h, followed by treatment with 1 μM nigericin for the indicated time. After removing the culture medium, cells were washed three times with PBS and fixed in 4% paraformaldehyde at room temperature for 30 min. The fixed cells were washed with PBS and blocked with 5% goat serum in PBS for 30 min at room temperature. The cells were then incubated with primary antibody overnight at 4 °C. After that, the cells were washed three times with PBS containing 0.1% Tween-20 (PBST), followed by secondary antibodies. Finally, antifade mounting medium with DAPI (Sigma-Aldrich, St Louis, MO, USA) was used to stain the cell nuclei at an indicated concentration. At least five fields per dish were randomly selected for observation, and the semiquantitative analysis of relative fluorescence intensity was analyzed by Image J 1.8.0 software (http://rsb.info.nih.gov/ij/, accessed on 1 February 2024). The antibodies used in this study are listed in Table S2.

### 2.13. Statistical Analysis

Statistical analysis was performed using IBM SPSS Statistics 22.0 (Chicago, IL, USA) and GraphPad Prism 9.0 (GraphPad Software, San Diego, CA, USA). The data of continuous variables fitting a normal distribution are presented as mean ± SD, and at least three independent experiments were performed in duplicate. The comparison of means of two independent samples was performed by two-tailed unpaired Student’s *t*-test. The comparisons of means of multiple groups were analyzed by one-way or two-way ANOVA followed by post hoc multiple comparisons. Data of non-continuous variables are presented as median (Q1, Q3), and the comparison of the medians of multiple independent samples was performed using the Kruskal–Wallis test, followed by Dunn’s multiple comparisons test. A *p* < 0.05 was considered as statistically significant.

## 3. Results

### 3.1. Ruscogenin Treatment Inhibits LPS/Nigericin-Induced Inflammatory Responses in THP-1 Cell and Improves the Intestinal Epithelial Barrier Function in Injured HIEC

**Figure 1:**
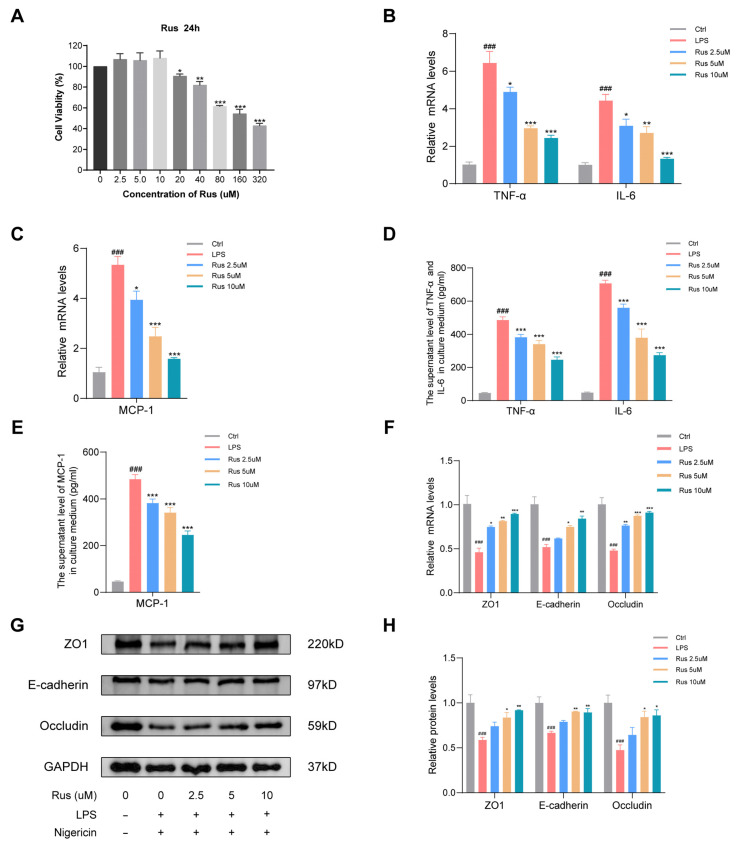
Ruscogenin treatment inhibited LPS-induced inflammatory responses in THP-1 cells and improves the intestinal epithelial barrier function in HIEC. (**A**) THP-1 cells were treated with DMSO or different concentrations of ruscogenin (2.5–320 μM) for 24 h, and the cell viability was determined by CCK-8 assay. (**B**,**C**) The mRNA expression of TNF-α, IL-6, and MCP-1 was detected with qPCR in THP-1 cells. (**D**,**E**) The supernatant levels of TNF-α, IL-6, and MCP-1 in the culture medium of THP-1 cells were detected via ELISA assay. (**F**) The mRNA expression of ZO1, E-cadherin, and occludin was detected with qPCR in HIEC. (**G**,**H**) The protein expression of ZO1, E-cadherin, and occludin in HIEC was analyzed by Western blotting and grayscale scanning analysis. Rus, ruscogenin; Ctrl, control group; LPS, LPS (100 ng/mL) exposure for 4 h group; Rus 2.5 μm, 2.5 μM ruscogenin pretreatment for 24 h prior to LPS exposure for 4 h, followed by treatment with 1 μM nigericin for 4 h group; Rus 5 μm, 5 μM ruscogenin pretreatment for 24 h prior to LPS exposure for 4 h, followed by treatment with 1 μM nigericin for 4 h group; Rus 10 μM, 10 μM ruscogenin pretreatment for 24 h prior to LPS exposure for 4 h, followed by treatment with 1 μM nigericin for 4 h group. Data are presented as the mean ± SD. ### *p* < 0.001 versus the control group; * *p* < 0.05, ** *p* < 0.01, and *** *p* < 0.001 versus the LPS group.

Macrophages are the main natural immune cells that play a vital role in inflammatory response. To detect the effect of ruscogenin in vitro, in this study, an in vitro macrophage inflammation model was established using THP-1 cells, wherein inflammation was induced by using LPS and nigericin. Firstly, the potential cytotoxicity of ruscogenin was evaluated by the CCK-8 assay. THP-1 cells were incubated with different concentrations of ruscogenin for 24 h. As shown in Figure 1A, ruscogenin treatment at concentrations below 20 μM for 24 h did not result in a decrease in the viability of THP-1 cells compared to the untreated control group. However, when the concentration of ruscogenin reached 20 μM, the cell viability of THP-1 was 9.3% lower than that of the control group (100% vs. 90.7%, *p* = 0.03), which means that slight toxicity was observed. Moreover, the cell viability of THP-1 decreased from 82.0% to 42.8% as the concentration of ruscogenin increased from 40 μM to 320 μM, exhibiting a significant dose-dependent cytotoxicity. Consequently, 2.5 μM, 5 μM, and 10 μM ruscogenin were selected as the low-, medium-, and high-dose groups, respectively, for further studies.

Inflammatory mediators are key players in the progression of IBD, contributing significantly to the advancement of the disease. To investigate whether ruscogenin possessed anti-inflammatory effects on THP-1 macrophages, THP-1 cells were treated with 2.5 μM, 5 μM, and 10 μM ruscogenin for 24 h. Subsequently, the cells were primed with 100 ng/mL LPS for 3 h in a serum-free medium, and then, the NLRP3 inflammasome was activated using 1 μM nigericin for 4 h. The supernatant levels of TNF-α, IL-6, and MCP-1 in the culture medium were detected via ELISA assay. After LPS stimulation, the concentrations of TNF-α, IL-6, and MCP-1 in the THP-1 cell supernatant surged to (485.5 ± 19.5), (708.0 ± 17.5), and (267.4 ± 8.4) pg/mL, respectively, which were at least three times higher than those in the control group (*p* < 0.001). Interestingly, ruscogenin treatment led to a significant decrease in the concentrations of these cytokine. Particularly, in the 10 μM ruscogenin treatment group, these cytokine concentrations decreased to (246.0 ± 17.3), (274.6 ± 14.2), and (75.9 ± 3.7) pg/mL, respectively (*p* < 0.001). Then, the mRNA expression of these inflammatory factors was detected with qPCR. Compared with the control group, the mRNA levels of TNF-α, IL-6, and MCP-1 were markedly elevated in the LPS group (Figure 1B–E). Interestingly, treatment with ruscogenin significantly reduced the mRNA levels of TNF-α, IL-6, and MCP-1, especially treatment with 10 μM ruscogenin (*p* < 0.001). These findings suggest that ruscogenin therapy may effectively mitigate the production of pro-inflammatory cytokines, thereby attenuating the inflammatory response.

To assess whether ruscogenin treatment could improve the intestinal epithelial barrier function, we conducted Western blotting analysis and qPCR to detect the expression of tight junction proteins in HIEC, including ZO1, E-cadherin, and occludin. HIEC were treated with 2.5 μM, 5 μM, and 10 μM ruscogenin for 24 h, followed by 100 ng/mL LPS for 3 h in serum-free medium and 1 μM nigericin for 4 h. As shown in Figure 1F–H, both the mRNA and protein levels of ZO1, E-cadherin, and occludin were significantly reduced after LPS/nigericin administration compared to the control group. When compared with the LPS/nigericin group, ruscogenin treatment effectively increased the expression levels of ZO1, E-cadherin, and occludin, thereby preserving the integrity of the intestinal barrier against the damage caused by LPS/nigericin treatment. Thus, these findings suggest that ruscogenin treatment inhibited LPS/nigericin-induced inflammatory responses in THP-1 cell and improved the intestinal epithelial barrier in injured HIEC.

### 3.2. Ruscogenin Suppresses the LPS/Nigericin-Induced Caspase-1-Dependent Pyroptosis in Macrophages

**Figure 2:**
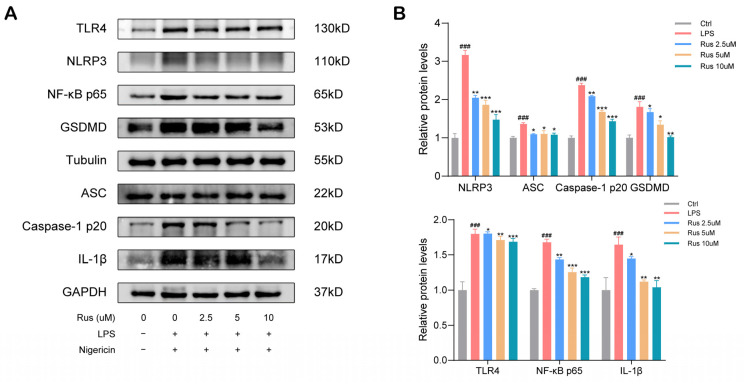

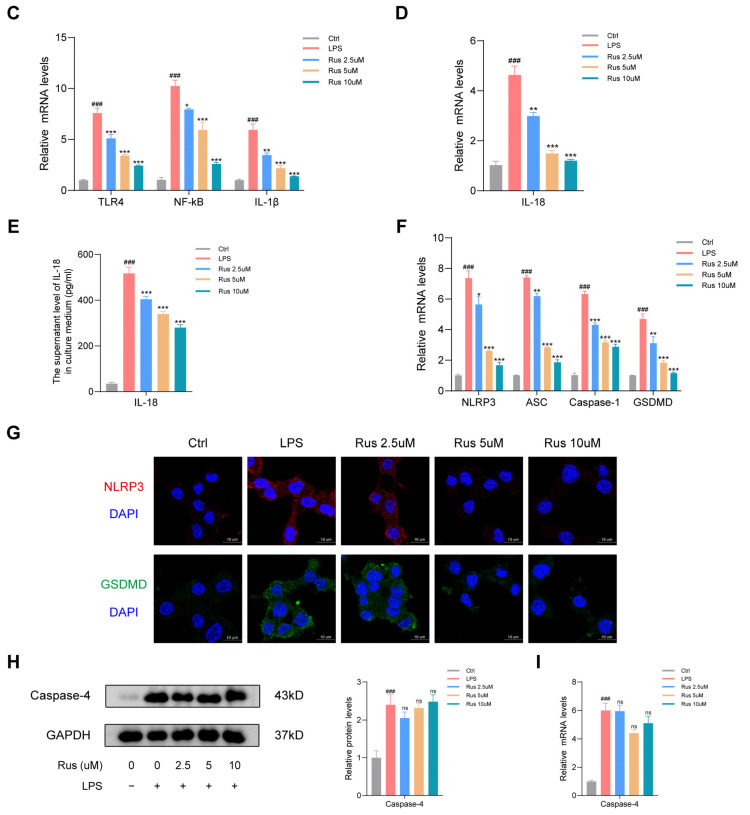
Ruscogenin suppresses the LPS/nigericin-induced caspase-1-dependent pyroptosis in macrophages. (**A**,**B**) The protein expressions of TLR4, NF-κB p65, NLRP3, ASC, caspase-1 p20, GSDMD, and IL-1β in THP-1 cells were analyzed by Western blotting and grayscale scanning analysis. (**C**) The mRNA expression levels of TLR4, NF-κB, and IL-1β in THP-1 cells were analyzed by qPCR. (**D**) The mRNA expression level of IL-18 was analyzed by qPCR. (**E**) The supernatant level of IL-18 in the culture medium of THP-1 cells was detected via ELISA assay. (**F**) The mRNA expressions levels of NLRP3, ASC, Caspase-1 p20, and GSDMD in THP-1 cells were analyzed by qPCR. (**G**) Immunofluorescence of NLRP3 and GSDMD in THP-1 cells. Scale bar: 10 µm. (**H**) After THP-1 cells were induced non-canonical pyroptosis using LPS, the protein expressions level of caspase-4 was analyzed by Western blotting. (**I**) After THP-1 cells were induced to non-canonical pyroptosis using LPS, the mRNA expressions level of caspase-4 was analyzed by qPCR. Rus, ruscogenin. Ctrl, control group; LPS, 100 ng/mL LPS; Rus 2.5 µm, 100 ng/mL LPS + 2.5 μM Rus; Rus 5 µm, 100 ng/mL LPS + 5 μM Rus; Rus 10 µm, 100 ng/mL LPS + 10 μM Rus. Data are presented as the mean ± SD. ns, not significant. ### *p* < 0.001 versus the control group; * *p* < 0.05, ** *p* < 0.01, and *** *p* < 0.001 versus the LPS group.

Next, we explored whether ruscogenin exerts its anti-inflammatory effect by suppressing the inflammasome activation and whether ruscogenin could suppress pyroptosis in LPS/nigericin-primed macrophages in vitro. Mature IL-1β and IL-18 are produced by cleavage of IL-1β precursor and IL-18 precursor by cleaved caspase-1 [13]. The human macrophage THP-1 cells were pretreated with ruscogenin, followed by LPS priming and nigericin induction, as described above. As shown in Figure 2A,B, compared with the control group, the Western blotting results showed that the expression of IL-1β was significantly elevated in the LPS/nigericin-induced group. Interestingly, pretreatment with ruscogenin significantly suppressed the expression of mature IL-1β. The same results were verified by qPCR (Figure 2C). The supernatants of the culture medium were collected, and the levels of mature IL-18 were analyzed by ELISA assay. Consistently, ruscogenin decreased the production of IL-18 (*p* < 0.01), while high concentrations of ruscogenin treatment had the biggest effects (Figure 2D,E). These results together indicate that ruscogenin treatment significantly inhibited LPS/nigericin-induced IL-1β and IL-18 release.

The NLRP3 inflammasome is an essential multiple protein complex comprised of NLRP3, ASC, and pro-caspase-1 that plays a crucial role in the development of canonical pyroptosis. The cleavage and activation of caspase-1 relies on the activation of NLRP3 inflammasome, and activated caspase-1 mediates the cleavage and activation of GSDMD, which is the executor of pyroptosis. We wondered whether ruscogenin might suppress the production of NLRP3, ASC, or caspase-1, so we detected the proteins extracted from the human macrophages THP-1 cells treated with ruscogenin. The results showed that the protein expression levels of NLRP3, ASC, caspase-1 p20, and GSDMD were upregulated in the LPS/nigericin-primed THP-1 cells but were significantly decreased after ruscogenin treatment, and 10 μM ruscogenin was more effective (Figure 2A,B). Additionally, the mRNA expression levels of NLRP3, ASC, caspase-1, and GSDMD were significantly higher in the LPS/nigericin-induced group than in the untreated control group by at least 300% (*p* < 0.001), whereas pretreatment with ruscogenin notably inhibited the expression of these genes (Figure 2C,F). The immunofluorescence staining showed that NLRP3 was elevated by LPS/nigericin stimulation, but the effect could be reversed by ruscogenin treatment (Figure 2G). Moreover, the results of immunofluorescence staining of GSDMD also indicated the suppressive functions of ruscogenin on LPS/nigericin-induced canonical pyroptosis (Figure 2G).

Furthermore, to investigate whether ruscogenin affects the non-canonical inflammasome pathway, we stimulated THP-1 with LPS to activate caspase-4 to mimic pyroptosis triggered by the non-canonical inflammasome pathway, according to the previous studies [25,26]. As shown in Figure 2H, the protein levels of caspase-4 were considerably increased after LPS treatment in comparison to the control group (*p* < 0.001). Nonetheless, ruscogenin treatment did not suppress the mRNA levels of caspase-4. In addition, the qPCR results indicated that ruscogenin treatment failed to rescue the increased mRNA levels of caspase-4 induced by LPS treatment (Figure 2I). This suggests that ruscogenin had little effect on the non-canonical pyroptosis pathway. Taken together, all these results indicated that ruscogenin could inhibit NLRP3 inflammasome activation and caspase-1-dependent pyroptosis in THP-1 cells.

### 3.3. Ruscogenin Ameliorates LPS/Nigericin-Induced Pyroptosis via Downregulating the Activation of NLRP3 Inflammasome and through TLR4/NF-κB Axis in Macrophages

**Figure 3:**
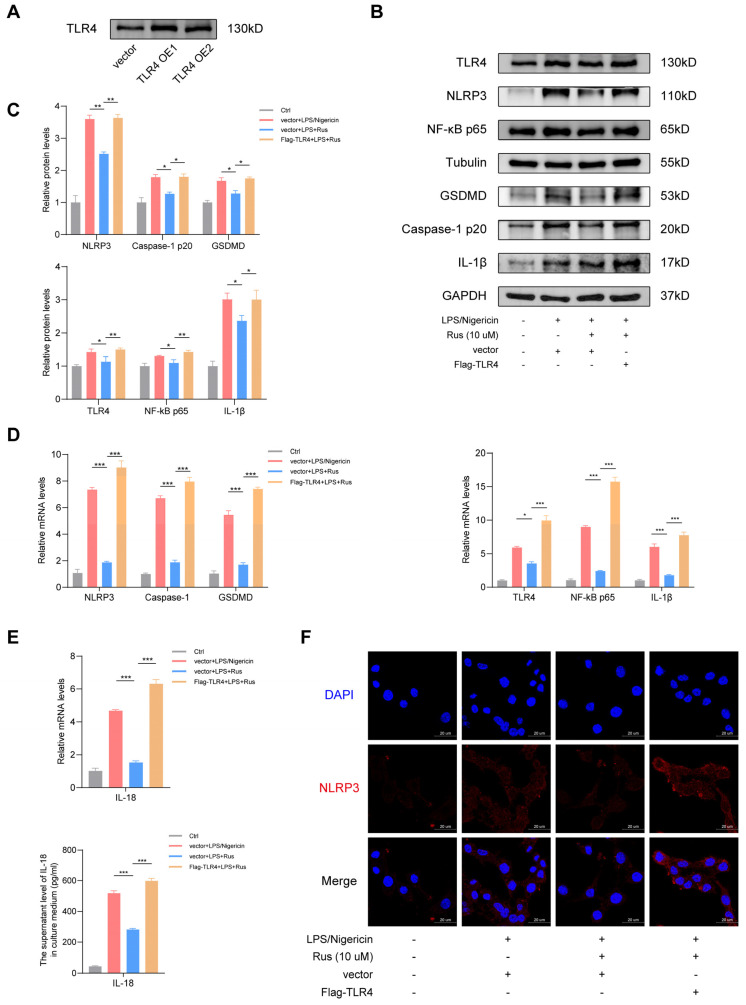
Ruscogenin ameliorates LPS/nigericin-induced pyroptosis via downregulating the activation of NLRP3 inflammasome and through TLR4/NF-κB axis in macrophages. (**A**) A overexpressed TLR4 THP-1 cell line were established using Flag-TLR4 plasmid and control plasmid. The expression level of TLR4 was detected by Western blotting. (**B**) The protein expressions of NLRP3 and IL-1β in THP-1 cells with overexpressed TLR4 were analyzed by western blotting and grayscale scanning analysis. (**C**) The protein expressions of TLR4, NF-κB, GSDMD, and caspase-1 p20 in THP-1 cells with overexpressed TLR4 were analyzed by Western blotting. (**D**) The mRNA expressions level of NLRP3, IL-18, and IL-1β were analyzed by qPCR. (**E**) The mRNA expressions level of TLR4, NF-κB, GSDMD, and caspase-1 p20 were analyzed by qPCR. (**F**) Immunofluorescence of NLRP3 in THP-1 cells with overexpressed TLR4. Scale bar: 20 µm. Rus, ruscogenin; Ctrl, normal THP-1 cells treated with DMSO; vector + LPS/nigericin, THP-1 cells transfected with control plasmid were treated with LPS/nigericin for 4 h; vector + LPS + Rus, THP-1 cells transfected with control plasmid were treated with 10 μM ruscogenin for 24 h, followed by LPS/nigericin 4 h; Flag-TLR4 + LPS + Rus, THP-1 cells transfected with TLR4-plasmid were treated with 10 μM ruscogenin for 24 h and then LPS/nigericin for 4 h. Data are presented as the mean ± SD. * *p* < 0.05, ** *p* < 0.01, and *** *p* < 0.001.

To explore the specific molecular mechanisms underlying the protective effects of ruscogenin on macrophages against inflammation and pyroptosis, we examined mRNA expression and protein levels of the upstream signaling molecules of TLR4–NF-κB signaling pathway. Western blotting and qPCR results showed that LPS/nigericin stimulation triggered the activation of TLR4 and NF-κB. Ruscogenin administration inhibited the expression of TLR4 and NF-κB, and the higher dose was more effective (Figure 2A,B). To confirm the effects of ruscogenin on LPS/nigericin-induced inflammation and pyroptosis in macrophages and to determine whether it plays a role through the TLR4/NF-κB signaling pathway, we established a THP-1 cell line overexpressing TLR4 using Flag-TLR4 plasmid and control plasmid. With a successful transfection (Figure 3A), we stimulated the transfected THP-1 cells with LPS and nigericin. The pyroptosis pathway-related protein expression was determined by Western blotting. As shown in Figure 3B–E, the results showed that compared with the control group, LPS/nigericin stimulation significantly elevated the protein and mRNA expression levels of TLR4, NF-κB p65, NLRP3, caspase-1, GSDMD, and IL-1β in THP-1 cells transfected control plasmid. Furthermore,10 μM ruscogenin treatment reduced the protein and mRNA level of TLR4, NF-κB p65, NLRP3, caspase-1, GSDMD, and IL-1β, but the expression levels of these pyroptosis pathway-related proteins and genes were markedly increased in TLR4-overexpressed THP-1 cells, even though they were treated with 10 μM ruscogenin. The results of NLRP3 immunofluorescence staining were identical to those of qPCR and Western blotting (Figure 3F). Collectively, these results indicating that ruscogenin ameliorated LPS/nigericin-induced inflammatory response and pyroptosis in macrophages, at least in part, through the TLR4/NF-κB signaling pathway.

### 3.4. Ruscogenin Treatment Attenuates DSS-Induced Colitis in Mice

**Figure 4:**
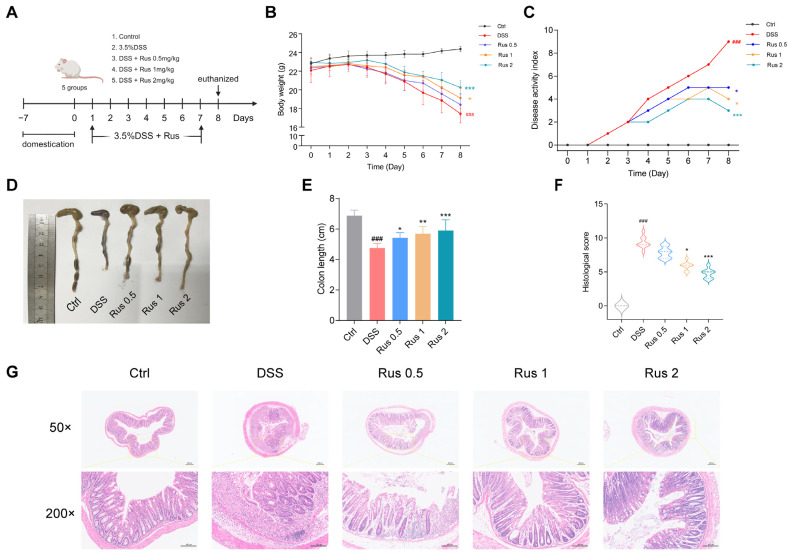
Ruscogenin attenuated the severity of DSS-induced colitis in mice. (**A**) Overview of the experimental protocol. C57BL/6 mice (*n* = 5–7 mice/group) were fed with distilled water or water containing 3.5% DSS and were intragastric injected with DMSO or ruscogenin (0.5/1/2 mg/kg) daily. Seven days later, all the water was changed to distilled water, and on the 8th day, the mice were euthanized, and their colon tissues were collected. (**B**) The body weight of each group. (**C**) The disease activity index of each group. (**D**,**E**) Colon tissues of mice were evaluated at the time of necropsy with representative macroscopic images. (**F**) Histological scores. At the center of each violin picture is a thick line that represents the median of the data. The upper and lower lines represent the first quartile and the third quartile. (**G**) Representative images of H&E staining of colon sections (magnification, 50× and 200×). The scale bars are 200 μm and 100 μm, respectively. DSS, Dextran sulfate sodium salt; Rus, ruscogenin; Ctrl, control group. DSS, 3.5% DSS group; Rus 0.5, 3.5% DSS + Rus 0.5 mg/kg group; Rus 1, 3.5% DSS + Rus 1 mg/kg group; Rus 2, 3.5% DSS + Rus 2 mg/kg group. Data in B and E are presented as the mean ± SD. Data in C are presented as the median. Data in F are presented as the median (Q1, Q3). ### *p* < 0.001 versus the control group; * *p* < 0.05, ** *p* < 0.01, and *** *p* < 0.001 versus the DSS group.

Next, we used 3.5% DSS to induce colitis in mice and evaluated the treatment effects of ruscogenin in vivo. The design of the experiment is shown in Figure 4A. In brief, the mice were treated with drinking water containing 3.5% DSS and with different doses of ruscogenin (0.5 mg/kg, 1 mg/kg, and 2 mg/kg) for 7 days. We evaluated colitis by closely monitoring the clinical symptoms of IBD, which include diarrhea, bloody feces, body weight loss, and colon shortening. As shown in Figure 4B, the results of two-way ANOVA showed that administration time, dose, and their interaction all had effects on the body weight of mice. We wanted to explore the difference in body weight among the groups of mice after 7 days of continuous administration. On day 8, the body weight of mice in the DSS group was significantly reduced compared with that in the control group (17.4 ± 0.9 g vs. 24.3 ± 0.2 g, *p* < 0.001), while 1 mg/kg ruscogenin treatment markedly attenuated the induced loss of body weight by 3.5% DSS (19.1 ± 1.1 g vs. 17.4 ± 0.9 g, *p* = 0.02), and high-dose ruscogenin (2 mg/kg) had a greater treatment effect on body weight loss (20.2 ± 0.7 g vs. 17.4 ± 0.9 g, *p* < 0.001). The median DAI score of the mice in the DSS group was higher than that of the control group on day 8 (*p* < 0.001). Interestingly, compared with DSS group, 0.5 mg/kg ruscogenin treatment decreased the median DAI score of mice (5.0 (4.0, 5.0) vs. 9.0 (8.5, 9.5), *p* < 0.001), while 2 mg/kg ruscogenin administration was more effective (3.0 (3.0, 3.75) vs. 9.0 (8.5, 9.5), *p* < 0.001) (Figure 4C). All the mice were euthanized on day 8, and the colon tissues and serum were collected for further analysis. The DSS-treated mice exhibited shorter and erythematous colons, both of which are characteristic indicators of colitis (4.7 ± 0.2 cm vs. 6.8 ± 0.3 cm, *p* < 0.001). However, these colonic abnormalities were effectively rescued by ruscogenin (Figure 4D,E). In addition, H&E staining of colon tissue sections is shown in Figure 4G. Compared with the control group, the H&E staining of the colon tissue in the DSS model group showed incomplete colonic mucosa, destruction and irregular arrangement of glandular structure, disappearance of crypt structure, and a large number of inflammatory cells having infiltrated the mucosa and submucosa, accompanied by edema. However, the administration of ruscogenin dramatically ameliorated the histopathological manifestations of colitis. The intestinal crypt and glandular structure of the mice treated with ruscogenin was more obvious than that of the DSS model group, and less inflammatory cell infiltration was observed in the mucosa, which was similar to that of the untreated mice. Thus, the median histological scores were also substantially lower in the ruscogenin treatment groups than in the DSS group, and high-dose ruscogenin showed greater efficacy (Figure 4F). All these results suggest that ruscogenin treatment significantly alleviated DSS-induced colitis in mice, effectively attenuating the clinical symptoms of colitis.

### 3.5. Ruscogenin Reduces the Inflammatory Response and Improves the Intestinal Barrier Function

**Figure 5:**
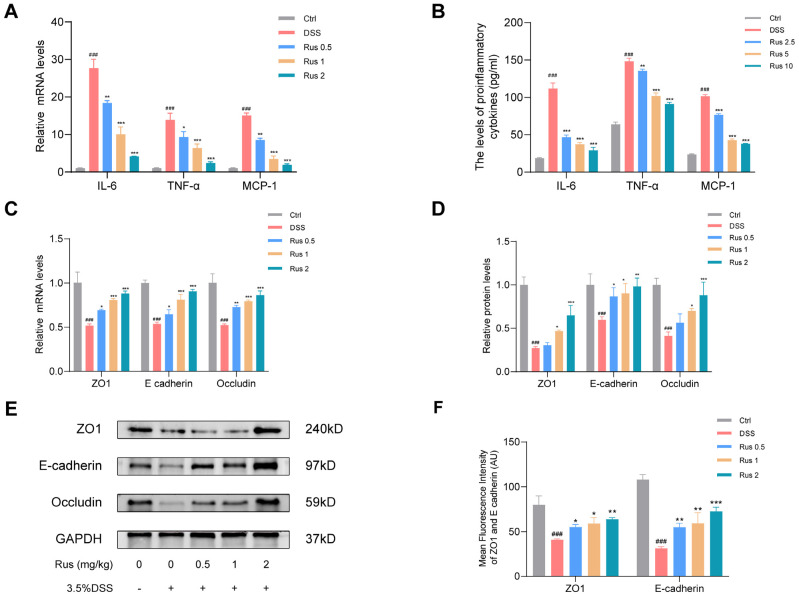

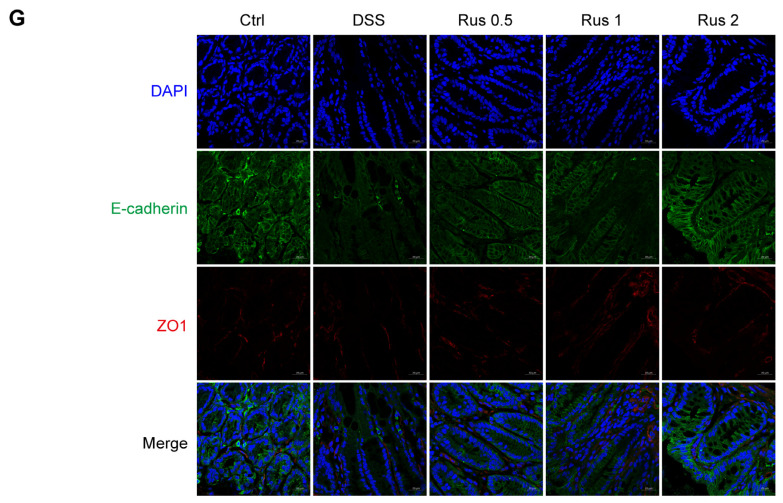
Effect of ruscogenin treatment on inflammatory cytokines and intestinal tight junction protein in DSS-induced mice colitis. (**A**) qPCR analysis of the mRNA expression level of inflammatory cytokines IL-6, TNF-α, and MCP-1 in colon tissues. (**B**) The levels of IL-6, TNF-α, and MCP-1 in colon tissues were detected by ELISA. (**C**) The mRNA expression levels of ZO1, E-cadherin, and occludin were analyzed by qPCR. (**D**,**E**) The protein expressions of ZO1, E-cadherin, and occludin in colon tissues were analyzed by Western blotting and grayscale scanning analysis. (**F**,**G**) The expressions of ZO1 and E-cadherin in colon tissue were analyzed by immunofluorescence staining. DAPI, blue; ZO1, red; E-cadherin, green. Scale bar: 20 µm. Rus, ruscogenin; Ctrl, control group; DSS, 3.5% DSS group; Rus 0.5, 3.5% DSS + Rus 0.5 mg/kg group; Rus 1, 3.5% DSS + Rus 1 mg/kg group; Rus 2, 3.5% DSS + Rus 2 mg/kg group. Data are presented as the mean ± SD. ### *p* < 0.001 versus the control group; * *p* < 0.05, ** *p* < 0.01, and *** *p* < 0.001 versus the DSS group.

To further evaluate the anti-inflammatory effects of ruscogenin, the expression of inflammatory cytokines IL-6, TNF-α, and MCP-1 were detected by qPCR (Figure 5A). Furthermore, the concentrations of IL-6, TNF-α, and MCP-1 in colon tissues were evaluated by ELISA (Figure 5B). All these results showed that the levels of these inflammatory cytokines were increased in the DSS group compared to that of in the control group, while ruscogenin treatment significantly reduced the levels of these inflammatory cytokines, especially in the Rus 2 group. Previous studies have demonstrated that epithelial barrier disruption is one of the main characteristics of IBD. Western blotting analysis, qPCR, and immunofluorescence staining were used to further determine whether ruscogenin could improve the intestinal mucosal barrier function in DSS-induced colitis. The mRNA expression levels of ZO1, E-cadherin, and occludin were significantly decreased in colon tissue in the DSS-administered mice compared to the control groups, but these changes were restored by ruscogenin treatment in a dose-dependent manner (Figure 5C). Moreover, Western blotting and immunofluorescence staining results were consistent with qPCR results and showed that DSS administration reduced the expression of E-cadherin, occludin, and ZO1, while ruscogenin treatment increased the level of these tight junction proteins in colon tissues from DSS-induced colitis mice, indicating that ruscogenin can improve intestinal barrier integrity in mice with colitis (Figure 5D–G). In short, the above results indicate that ruscogenin attenuates inflammatory response in DSS-induced colitis mice, and ruscogenin prevents the DSS-induced colitis by regulating tight junction proteins and improving the intestinal barrier function.

### 3.6. Ruscogenin Alleviates DSS-Induced Colitis in Mice by Inhibiting NLRP3 Inflammasome Activation and Caspase-1-Dependent Pyroptosis

**Figure 6:**
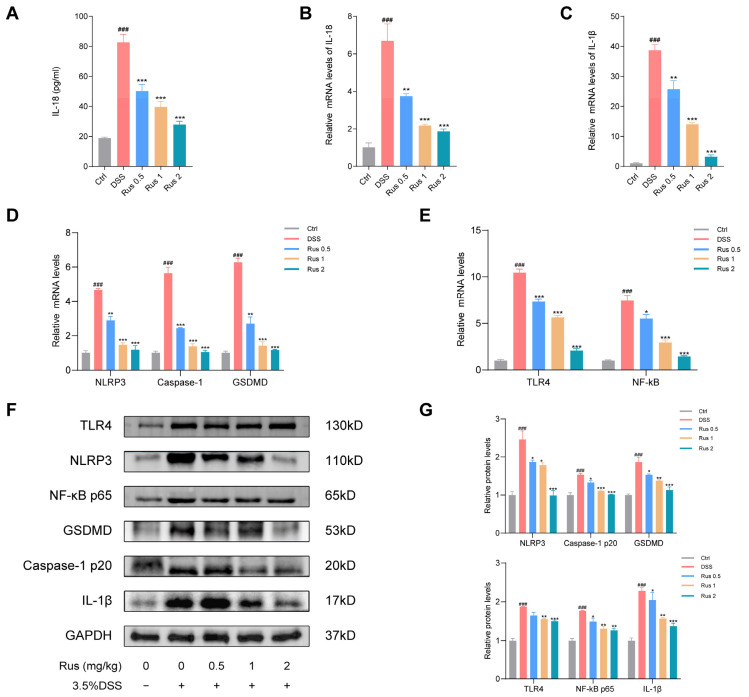
Ruscogenin treatment inhibits the activation of NLRP3 inflammasome and caspase-1-dependent pyroptosis in DSS-induced mice colitis. (**A**) The levels of IL-18 in colon tissues were detected by ELISA. (**B**) The mRNA expressions level of IL-18 was analyzed by qPCR. (**C**) The mRNA expressions level of IL-1β was detected by qPCR. (**D**,**E**) The mRNA expression levels of NLRP3, caspase-1 p20, GSDMD, TLR4, and NF-κB were analyzed by qPCR. (**F**,**G**) The protein expression levels of NLRP3, caspase-1 p20, GSDMD, TLR4, NF-κB p65, and IL-1β in colon tissues were analyzed by Western blotting and grayscale scanning analysis. Rus, ruscogenin; Ctrl, control group; DSS, 3.5% DSS group; Rus 0.5, 3.5% DSS + ruscogenin 0.5 mg/kg group; Rus 1, 3.5% DSS + ruscogenin 1 mg/kg group; Rus 2, 3.5% DSS + ruscogenin 2 mg/kg group. Data are presented as the mean ± SD. ### *p* < 0.001 versus the control group; * *p* < 0.05, ** *p* < 0.01, and *** *p* < 0.001 versus the DSS group.

Previous reports have demonstrated a significant correlation between the activity of NLRP3 inflammasomes and the development as well as the pathogenesis of IBD. They have also found that IBD patients exhibit high expression of pyroptosis-related factors both at the mRNA level and protein level [12]. Based on the findings from in vitro experiments indicating that ruscogenin exhibited the ability to inhibit inflammation and pyroptosis, we proceeded to explore whether ruscogenin could alleviate DSS-induced colitis by inhibiting the NLRP3 inflammasome and pyroptosis via the TLR4–NF-κB signaling pathway. We conducted qPCR, ELISA, Western blotting, and immunofluorescence staining to measure the expression of pyroptosis-related molecular levels. As shown in Figure 6A–D, the mRNA expression levels of NLRP3, caspase-1, GSDMD, IL-18, and IL-1β of the mice in the DSS group were upregulated after DSS administration, but these indicators were significantly decreased after the mice were treated with ruscogenin, and 2 mg/kg ruscogenin administration was more obvious (*p* < 0.001). All these results were consistent with the results of Western blotting, showing that the protein expression levels of NLRP3, caspase-1, GSDMD, and IL-1β in colon tissues of the mice were markedly reduced in both the Rus 1 group and Rus 2 group compared with the DSS model group (Figure 6E–G). Consistent with the in vitro results, ruscogenin treatment inhibited the expression of TLR4 and NF-κB, and the higher dose was more effective (Figure 6E–G). Taken together, these results highlight that the protective effect of ruscogenin on DSS-induced colitis was associated with the attenuation of NLRP3 inflammasome activation and caspase-1-dependent pyroptosis by inhibiting the TLR4/NF-κB pathway.

## 4. Discussion

In this study, we used a DSS-induced mice colitis model and LPS/nigericin-induced THP-1 macrophages to illustrate ruscogenin’s capacity to inhibit the inflammatory response and pyroptosis. Ruscogenin significantly inhibited LPS/nigericin-induced canonical pyroptosis in human monocytic leukemia cell THP-1 but had minimal impact on LPS-induced non-canonical pyroptosis in THP-1 cells. Moreover, it displayed a mitigating effect on DSS-induced colitis in vivo. In terms of the underlying mechanism, the TLR4/NF-κB pathway plays a vital role in the activation of NLRP3 inflammasome and caspase-1-dependent canonical pyroptosis instead of caspase-1-independent non-canonical pyroptosis [27]. By using a TLR4 overexpression plasmid, we found that ruscogenin treatment potentially alleviates DSS-induced colitis in mice by inhibiting NLRP3 inflammasome activation and canonical pyroptosis, and the protective effect may be related to the suppression of the TLR4/NF-κB signaling pathway. Through a combination of in vitro and in vivo experiments, we successfully demonstrated the therapeutic effects and potential mechanism of ruscogenin.

The doses of ruscogenin used for animal experiments in our study were 0.5, 1, and 2 mg/kg, based on the previous study [18,19]. The DSS-induced mice colitis model is a well-established and widely used model for studying the pathogenesis and drug efficacy of human UC [28]. The therapeutic efficacy of ruscogenin is manifested in the significant reduction of UC symptoms like diarrhea and rectal bleeding in mice treated with DSS. Additionally, ruscogenin administration led to the reversal of weight loss and a decrease in DAI scores. Colonic shortening, a common symptom induced by DSS, was notably alleviated with ruscogenin treatment. Histopathological evaluations revealed that the mice in the DSS group exhibited irregularities in the submucosa and crypt structures, but these abnormalities were significantly improved in the groups treated with ruscogenin. These findings indicate that ruscogenin may be a good candidate drug for the treatment of UC.

Recent research has indicated a strong correlation between the expression of proinflammatory molecules and intestinal epithelial barrier disruption and the occurrence and progression of UC, contributing to intestinal immune system disorders [29]. Intestinal epithelial cells typically serve as the first line of defense against intestinal diseases. The physical function of the intestinal barrier is damaged due to the accumulation of pyroptosis in intestinal epithelial cells and the dysfunction of tight junction proteins, which consequently leads to an increase in intestinal permeability [30]. Colonic inflammation can occur due to the presence of intestinal pathogens invasion as well as the breakdown of tight junctions caused by a continuous cycle of releasing both pro-inflammatory and anti-inflammatory factors [31]. This process triggers the adaptive immune system, leading to the activation of antigen-presenting cells and the transformation of naive T cells into effector T cells like Th1, Th2, Th17, and natural killer T cells [32]. These cells generate a variety of proinflammatory cytokines, including IL-1β, which activate macrophages to release a large number of cytokines, such as TNF-α, IL-6, and MCP-1, causing tissue damage. TNF-α, an early endogenous mediator in major inflammatory diseases, can disrupt the function of the intestinal barrier. IL-1β and MCP-1 also play a vital role in regulating inflammation, particularly in the early stages, leading to colonic inflammation. Recent studies have shown that advanced therapies targeting specific inflammatory pathways can treat moderate-to-severe UC [29,33]. In this study, our findings showed a significant increase in the levels of TNF-α, IL-6, and MCP-1 after the administration of DSS or LPS/nigericin. Interestingly, ruscogenin treatment reduced the levels of these proinflammatory cytokines in both colon tissues and culture medium supernatant as well as the expression of their corresponding mRNAs. Moreover, we confirmed the disruption of tight junction proteins in DSS-treated mice, while administration of ruscogenin significantly improved the intestinal barrier function by increasing ZO1, occludin, and E-cadherin expressions during epithelial damage. Therefore, these data demonstrate that ruscogenin treatment alleviates DSS-induced colitis by decreasing the secretion of inflammatory factors and repairing the expression of tight junctions and their associated proteins.

Pyroptosis serves as an immune defense mechanism against invading pathogens, characterized by cell membrane disruption and the secretion of inflammatory cytokines. However, when pyroptosis becomes uncontrolled due to overactive inflammasomes, it can lead to significant cell membrane damage and an excessive release of inflammatory factors, disrupting homeostasis and potentially leading to the development of various diseases. Recent studies have shown that pyroptosis is involved in the pathogenesis of UC [11,34]. Thus, the control of inflammatory response and pyroptosis of macrophage prevents extensive tissue damage in UC. Regarding LPS/nigericin-induced inflammatory and canonical pyroptosis, it is well known that TLR4 initiates the immune response by recognizing LPS. The most important pathway for LPS activation is the MyD88-dependent pathway [35]. First, after LPS stimulation, the intracellular Toll/IL-1 receptor structural domain of TLR4 binds to MyD88, which then induces NF-κB signaling, causing elevated expression of NLRP3, pro-IL-18, and pro-IL-1β. Then, nigericin indirectly activates NLRP3, leading to NLRP3 inflammatory complex assembly and caspase-1 activation. As a downstream mediator of the TLR4 signaling pathway, NF-κB is a transcription factor involved in various pathophysiological processes, including inflammation, pyroptosis, and immune processes [14,36]. Ruscogenin showed prominently anti-inflammatory activity in animal experimental models of cerebral ischemic injury, acute lung injury, and hepatic injury [17,18,20,37]. A previous study indicated that the possible molecular mechanism by which ruscogenin inhibits the inflammatory response is mainly related to the inhibition of the NF-κB signaling pathway [17]. Wu et al. found that ruscogenin ameliorated LPS-induced apoptosis of pulmonary endothelial cells mainly by targeting TLR4 [19]. Interestingly, another study conducted by Wang et al. also suggested that ruscogenin effectively alleviates particulate matter-induced acute lung injury probably by inhibiting TLR4/MyD88 signaling pathway. TLR4 might be vital for particulate matter to initiate pulmonary lesions and for ruscogenin to exert treatment effect on lung injury [18]. A newly published study reported that ruscogenin attenuates SARS-CoV-2 E protein-induced pyroptosis by inhibiting NLRP3 inflammasome activation [38]. Consistently, in this study, our novel data showed that ruscogenin effectively inhibited caspase-1-dependent pyroptosis via inhibiting the activation of NLRP3 inflammasome. Moreover, to exclude that ruscogenin affects pyroptosis through the caspase-4-dependent non-canonical inflammasome pathway, we analyzed the effect of ruscogenin on the non-canonical inflammasome pathway by stimulating HIEC with LPS alone. The results demonstrated that ruscogenin had little effect on the caspase-4-dependent non-canonical inflammasome pathway. Further results found that ruscogenin failed to ameliorate LPS/nigericin-induced pyroptosis and NLRP3 inflammasome activation when overexpressing TLR4, indicating that TLR4 is a potential target of ruscogenin, which is consistent with the findings of other studies mentioned above. Thus, our findings confirmed that this protective effect of ruscogenin may be related to TLR4/NF-κB pathway inhibition. It might provide a promising strategy for the treatment of UC patients. In short, we successfully demonstrated the therapeutic effects and potential mechanism of ruscogenin in the treatment of UC.

Aside from these findings, several potential limitations of this present study exist. For instance, the results of this study showed that ruscogenin inhibited the TLR4/NF-κB pathway, but the mediator involved is still unclear and needs to be explored. Furthermore, although we confirmed the finding that ruscogenin inhibits pyroptosis and thereby alleviates colitis through the TLR4/NF-κB pathway, transgenic mouse experiments are necessary to verify this pathway before using ruscogenin in IBD patients. Finally, ruscogenin restored the intestinal barrier in UC by reducing inflammatory responses and inhibiting pyroptosis. However, ruscogenin may also improve intestinal barrier function and inflammation through other means, and whether ruscogenin could protect the intestinal barrier disruption through other related signaling pathways remains unclear and deserves further research.

## 5. Conclusions

In summary, this study elucidated the efficient protective role of ruscogenin against DSS-induced colitis and demonstrated that ruscogenin alleviates DSS-induced colitis in mice by inhibiting NLRP3 inflammasome activation and caspase-1-dependent canonical pyroptosis, probably due to the suppression of the TLR4/NF-κB signaling pathway. These findings might provide new insights and a solid foundation for further exploration into the therapeutic potential of ruscogenin in the treatment of IBD.

## Data Availability

The original contributions presented in the study are included in the article/Supplementary Material. Further inquiries can be directed to the corresponding author.

## Supplementary Materials

The following supporting information can be downloaded at: https://www.mdpi.com/article/10.3390/biomedicines12050989/s1, Table S1. Primers used in qPCR; Table S2. Antibodies.

## Abbreviations

IBD, inflammatory bowel disease; UC, ulcerative colitis; Rus, ruscogenin; NLRP3, NOD-like receptor pyrin domain-containing protein 3; ZO1, Zonula occludens 1; TLR4, Toll-like receptors 4; PRR, pattern recognition receptor; PAMP, pathogen-related molecular pattern; DAMP, damage-related molecular pattern; IL-1β, interleukin-1β; GSDMD, gasdermin D; LPS, lipopolysaccharide; H&E, hematoxylin and eosin; DAI, disease activity index; CCK-8, cell counting kit-8; qPCR, quantitative real-time polymerase chain reaction.
