# Supplementary material for: Ruscogenin Attenuates Ulcerative Colitis in Mice by Inhibiting Caspase-1-Dependent Pyroptosis via the TLR4/NF-κB Signaling Pathway
Source: Biomedicines. 2024 Apr 30;12(5):989. doi: 10.3390/biomedicines12050989 (PMC11117655; doi:10.3390/biomedicines12050989)
Supplement: Supplementary file 1 [file biomedicines-12-00989-s001.zip › biomedicines-2924760-supplementary.pdf]

Additional file S1

Table S1. Primers used in qPCR

Table S2. Antibodies

Table S1. Primers used in qPCR

Gene	Forward Primer (5'→3')	Reverse Primer (5'→3')
Mouse TNF-α	CGACGTGGAACTGGCAAGAA	GGACCGATCACCCCGAAG
Mouse IL-6	CTGCAAGAGACTTCCATCCAG	AGTGGTATAGA-CAGGTCTGTTGG
Mouse MCP-1	CTGTGCTGACCCCAAGAAGG	AGGTGGTTGTGGAAAAGGTAGTG
Mouse IL-1β	ATGCCACCTTTTGACAGTGATG	TGATGTGCTGCTGCGAGATT
Mouse IL-18	AAGGACACTTTCTTGCTTGCC	GCCTCGGGTATTCTGTTATGG
Mouse NLRP3	ATTACCCGCCCGAGAAAGG	TCGCAGCAAAGATCCACACAG
Mouse ASC	CTTGTCAGGGGATGAACTCAAAA	GCCATACGACTCCAGATAGTAGC
Mouse GSDMD	AGGTGACAGAAAAGCAGTGGG	GCTGGGCTGGTCCTGTAAAAT
Mouse Caspase-1	ACATCTTTCTCCGAGGGTTGG	TCTGGGCAGGCAGCAAAT
Mouse GSDMD	AGGTGACAGAAAAGCAGTGGG	GCTGGGCTGGTCCTGTAAAAT
Mouse TLR4	GCCTTTCAGGGAATTAAGCTCC	GATCAACCGATGGACGTGTAAA
Mouse NF-κB	GGAGGCATGTTCGGTAGTGG	CCCTGCGTTGGATTTCGTG
Mouse ZO1	GCCGCTAAGAGCACAGCAA	TCCCCACTCTGAAAATGAGGA
Mouse E-cadherin	CAGGTCTCCTCATGGCTTTGC	CTTCCGAAAAGAAGGCTGTCC
Mouse Occludin	TGAAAGTCCACCTCCTTACAGA	CCGGATAAAAAGAGTACGCTGG
Mouse GAPDH	AGGTCGGTGTGAACGGATTTG	TGTAGACCATGTAGTTGAGGTCA
Human TNF-α	CCTCTCTCTAATCAGCCCTCTG	GAGGACCTGGGAGTAGATGAG
Human IL-6	CGGACAGCTTGAACAGAATGT	ACCATCCCACTCACACCTCA
Human MCP-1	CAGCCAGATGCAATCAATGCC	TGGAATCCTGAACCCACTTCT
Human IL-1β	ATGATGGCTTATTACAGTGGCAA	GTCGGAGATTCGTAGCTGGA
Human IL-18	TCTTCATTGACCAAGGAAATCGG	TCCGGGGTGCATTATCTCTAC

Human NLRP3	CGTGAGTCCCATTAAGATGGAGT	CCCGACAGTGGATATAGAACAGA
Human ASC	TGGATGCTCTGTACGGGAAG	CCAGGCTGGTGTGAAACTGAA
Human Caspase-1	TTGAAGGACAAACCGAAGGTG	GTGGAAGAGCAGAAAGCGATAA
Human GSDMD	GTGTGTCAACCTGTCTATCAAGG	CATGGCATCGTAGAAGTGGAAG
Human Caspase-4	CAGCACAATGGGCTCTATCTTC	GGACAGTCGTTCTATGGTGGG
Human TLR4	CCTCGGCGGCAACTTCATAA	AGAGCGGATCTGGTTGTACTG
Human NF-κB	GGTGCGGCTCATGTTTACAG	GATGGCGTCTGATACCACGG
Human ZO1	CAACATACAGTGACGCTTCACA	CACTATTGACGTTTCCCCACTC
Human E-cadherin	CGAGAGCTACACGTTCACGG	GGGTGTCGAGGGAAAAATAGG
Human Occludin	GGCACCTGCATACTCACCC	CTGGGAGAGCAACTCATCCTC
Human GAPDH	ACAACTTTGGTATCGTGGAAGG	GCCATCACGCCACAGTTTC

Table S2. Antibodies

Antibodies	Application	Cat.	Company
GAPDH	WB (1:1000)	#2118S	CST
IL-1β	WB (1:1000)	ab254360	Abcam
β-Actin	WB (1:1000)	#4970S	CST
NLRP3	WB (1:1000) , IF (1:200)	ab263899	Abcam
Caspase-1	WB (1:1000)	ab179515	Abcam
ASC	WB (1:1000)	ab309497	Abcam
GSDMD	WB (1:1000), IF (1:200)	ab219800	Abcam
TLR4	WB (1:1000)	66350-1-Ig	Proteintech
NF-kB p65	WB (1:1000)	ab32536	Abcam
Caspase-4	WB (1:1000)	ab238124	Abcam
ZO1	WB (1:1000), IF (1:100)	ab276131	Abcam
E-cadherin	WB (1:1000), IF (1:1000)	ab231303	Abcam
Occludin	WB (1:1000), IF (1:100)	ab216327	Abcam
TLR4	WB (1:1000)	ab13556	Abcam
HRP Goat Anti-Rabbit IgG H&L	WB (1:2000)	#7077S	CST
HRP Goat Anti-Mouse IgG H&L	WB (1:2000)	#7076S	CST
Goat Anti-Rabbit IgG H&L (Alexa Fluor® 488 Conjugate)	IF (1:500)	ab150077	Abcam
Goat Anti-Rabbit IgG H&L (Alexa Fluor® 647 Conjugate)	IF (1:500)	ab150075	Abcam
Goat Anti-Rabbit IgG H&L (Alexa Fluor® 594 Conjugate)	IF (1:500)	A11037	Invitrogen
Goat Anti-Mouse IgG H&L (Alexa Fluor® 488 Conjugate)	IF (1:500)	A11001	Invitrogen

CST, Cell Signaling Technology
